# Ca^2+^/Calmodulin-Dependent Protein Kinase II Enhances Retinal Ganglion Cell Survival But Suppresses Axon Regeneration after Optic Nerve Injury

**DOI:** 10.1523/ENEURO.0478-23.2024

**Published:** 2024-03-21

**Authors:** Xin Xia, Caleb Shi, Christina Tsien, Catalina B. Sun, Lili Xie, Ziming Luo, Minjuan Bian, Kristina Russano, Hrishikesh Singh Thakur, Larry I. Benowitz, Jeffrey L. Goldberg, Michael S. Kapiloff

**Affiliations:** ^1^Department of Ophthalmology, Byers Eye Institute, Mary M. and Sash A. Spencer Center for Vision Research, Stanford University School of Medicine, Palo Alto, California 94034; ^2^Department of Neurosurgery, F.M. Kirby Neurobiology Center, Boston Children’s Hospital, Boston, Massachusetts 02115; ^3^Department of Neurosurgery and Ophthalmology, Harvard Medical School, Boston, Massachusetts 02115

**Keywords:** CaMKII, glaucoma, neuroprotection, optic nerve injury, retinal ganglion cells

## Abstract

Neuroprotection after injury or in neurodegenerative disease remains a major goal for basic and translational neuroscience. Retinal ganglion cells (RGCs), the projection neurons of the eye, degenerate in optic neuropathies after axon injury, and there are no clinical therapies to prevent their loss or restore their connectivity to targets in the brain. Here we demonstrate a profound neuroprotective effect of the exogenous expression of various Ca^2+^/calmodulin-dependent protein kinase II (CaMKII) isoforms in mice. A dramatic increase in RGC survival following the optic nerve trauma was elicited by the expression of constitutively active variants of multiple CaMKII isoforms in RGCs using adeno-associated viral (AAV) vectors across a 100-fold range of AAV dosing in vivo. Despite this neuroprotection, however, short-distance RGC axon sprouting was suppressed by CaMKII, and long-distance axon regeneration elicited by several pro-axon growth treatments was likewise inhibited even as CaMKII further enhanced RGC survival. Notably, in a dose-escalation study, AAV-expressed CaMKII was more potent for axon growth suppression than the promotion of survival. That diffuse overexpression of constitutively active CaMKII strongly promotes RGC survival after axon injury may be clinically valuable for neuroprotection per se. However, the associated strong suppression of the optic nerve axon regeneration demonstrates the need for understanding the intracellular domain- and target-specific CaMKII activities to the development of CaMKII signaling pathway-directed strategies for the treatment of optic neuropathies.

## Significance Statement

In optic neuropathies such as glaucoma, the prevention of retinal ganglion cell (RGC) loss and the regeneration of RGC axons are the two main goals of clinical therapy. The expression of constitutively activated CaMKII in RGCs using gene therapy vectors is highly efficacious in promoting neuroprotection after optic nerve injury. However, the new data presented herein demonstrate that this neuroprotection is associated with the suppression of axon regeneration. This latter effect should be considered in the development of CaMKII-directed therapies and underscores the need for further investigation into the mechanisms conferring the functions of CaMKII in the RGC.

## Introduction

The failure of central nervous system (CNS) neurons to survive and regenerate axons after injury or in degenerative disease remains a major problem in basic and translational neuroscience. For example, optic neuropathies including glaucoma, ischemic optic neuropathy, optic neuritis, and trauma to the optic nerve comprise some of the leading causes of irreversible blindness and result in the death of retinal ganglion cells (RGCs; Chang and Goldberg, 2012). As the sole output neurons for the retina, RGCs transmit all the visual information from the retina via the optic nerve to the lateral geniculate, superior colliculus, suprachiasmatic, and other nuclei of the brain. Thus, preservation of RGCs and regeneration of damaged RGC axons are important goals for the treatment of optic neuropathies, and understanding the molecular and cellular regulation of these processes remains an important fundamental issue.

Like other CNS neurons, RGC regenerative failure has been linked to molecular pathways including transcriptional regulators (Moore et al., 2009; Watkins et al., 2013; Apara and Goldberg, 2014; Chang et al., 2021; Cheng et al., 2022), second-messenger signaling pathways (Boczek et al., 2019), and signaling kinases and phosphatases (Park et al., 2008; Lorber et al., 2009; Kunzevitzky et al., 2010; Galvao et al., 2018). Remarkably, the expression of the activated forms of the multifunctional Ca^2+^/calmodulin-dependent protein kinase II (CaMKII) was recently reported to confer RGC neuroprotection in mouse models of optic nerve injury, glaucoma, and NMDA excitotoxicity at a level not seen before in animal models (Guo et al., 2021). Consequently, such robust RGC survival makes CaMKII a leading target for therapeutic intervention in optic neuropathies. CaMKII is a dodecameric serine/threonine protein kinase expressed by the four alternatively spliced genes *Camk2a-d* (expressing CaMKII α, β, γ, and δ, respectively), including >40 commonly detected isoforms (Sloutsky and Stratton, 2021). Essentially all RGCs express CaMKII (Terashima et al., 1994; Tetenborg et al., 2017), although studies in primates suggest that RGC levels of the best characterized isoform CaMKIIα vary widely among different RGC subtypes (Baldicano et al., 2022). Prior research has occasionally identified differential functions of the CaMKII isoforms. For example, in vitro siRNA-mediated depletion of nuclear CaMKIIαB worsened RGC survival after the glutamate exposure (Fan et al., 2007), whereas CaMKIIγ and CaMKIIδ promote cortical neuron survival in vitro during oxygen/glucose deprivation and reoxygenation (Ye et al., 2019). Furthermore, the paradoxical effects of CaMKII isoforms have been described, as inhibition of CaMKII activity can be beneficial in stroke, and CaMKII inhibitors have been proposed as therapeutics promoting neuroprotection in cerebrovascular disease (Deng et al., 2017; Zhang et al., 2021).

Given that two constitutively active isoforms of CaMKII (α and β) demonstrated a remarkable effect on RGC neuroprotection (Guo et al., 2021) and the potential translatable value of this approach, there is a strong motivation to confirm and extend this finding, explore the isoform specificity, and determine the concurrent effects on axon regeneration. Here we used a well-established model of optic nerve trauma (Cameron et al., 2020) to study RGC survival and axonal regeneration. Our findings in two independent labs confirm the effects of constitutively active CaMKII on RGC neuroprotection and extend those data by exploring dose–response relationships and the function of multiple isoforms of CaMKII. Importantly, however, we uncover a significant inhibition of RGC axon sprouting and long-distance regeneration in the optic nerve and demonstrate that this inhibition is dominant over several previously identified promoters of optic nerve axon regeneration.

## Materials and Methods

### Animals

All animal procedures were performed in accordance with the Association for Research in Vision and Ophthalmology Statement for the Use of Animals in Ophthalmic and Vision Research and approved by the Institutional Animal Care and Use Committee at Stanford University (Administrative Panel on Laboratory Animal Care) or Boston Children's Hospital. Wild-type 129X1/SvJ, 4–6-week-old male and female mice from the Jackson Laboratory (RRID:IMSR_JAX:000691) were used for CaMKII isoform experiments performed at Stanford University. Wild-type 129S1/SvlmJ, 5–10-week-old male and female mice from the Jackson Laboratory (RRID:IMSR_JAX:002448) were used for experiments involving CaMKIIα T286D expression in combination with proregenerative treatments performed at Boston Children's Hospital.

### Viral vectors

T286D (α) or T287D (β, γ, δ) mutants of CaMKII isoforms contain a phospho-mimetic amino acid substitution that renders the protein Ca^2+^/calmodulin independent, allowing the study of CaMKII function independently of upstream stimuli (Bhattacharyya et al., 2020). Adeno-associated virus type 2 (AAV2)-expressing CaMKIIα T286D and green fluorescent protein (GFP) control under the control of a ubiquitous CMV early enhancer - modified chicken β-actin (CAG) promoter were kindly provided by Guo et al. (2021). AAV2 expressing ciliary neurotrophic factor (CNTF) was as previously described (Xie et al., 2021).

New AAV transfer plasmids were constructed that express Flag-tagged constitutively active CaMKII mutant proteins under the control of a −1,320 to +110 base pair mouse γ-synuclein (*Sncg*) promoter (C57BL/6J chromosome 14 NCBI Reference Sequence: NC_000080.7) previously shown to confer RGC-selective expression (Wang et al., 2020), a 123 bp cytomegalovirus early enhancer fragment, an optimized truncated version of the woodchuck hepatitis virus posttranscriptional regulatory element, and a bovine growth hormone polyadenylation signal. Rat CaMKII cDNAs containing a T286D (α) or T287D (β, γ, δ) substitution mutation were based on the following NCBI sequences: CaMKIIα, NM_012920.1; CaMKIIαB, XM_039096592.1; CaMKIIβ, NM_021739.2; CaMKIIβ’, XM_006251176.4; CaMKIIβe, XM_017599071.2; CaMKIIβH, XM_017599072.2; CaMKIIγ X1, XM_008770486.3; CaMKIIδA, XM_017590605.2; and CaMKIIδB, XM_039101692.1. An AAV transfer plasmid to express GFP was similarly designed. Transfer plasmids were constructed by VectorBuilder. Complete plasmid and AAV sequences are available upon request.

The *Sncg* promoter AAVs were prepared by HEK293 triple-plasmid transfection and cesium chloride density gradient centrifugation by the Stanford Department of Ophthalmology AAV Core as previously described (Wang et al., 2020), were single-stranded, and contained serotype 2 triple mutant (AAV2tm) Y444/500/730F capsid that has increased penetrance of retinal cell delivery compared with wild-type AAV2 capsid (Petrs-Silva et al., 2011). AAV2tm stocks were 10^12^–10^13^ vg/ml, as determined by quantitative alkaline gel electrophoresis.

### Intravitreal injections

To test the effects of constitutively active CaMKII overexpression, beginning 2–3 weeks before optic nerve injury, we intravitreally injected the wild-type mice with 1.5 µl AAV in PBS through the sclera 1 mm below the limbus by foot pedal-controlled Picospritzer III–Intracellular Microinjection Dispense Systems (Parker Hannifin). Other intravitreally injected reagents included zymosan (12.5 mg/ml, Sigma-Aldrich), oncomodulin (Ocm, 30 ng/ml), stromal-derived factor 1 (SDF-1; 100 ng/ml, Chemicon), the nonhydrolyzable cAMP analog CPT-cAMP (50 μM, Sigma-Aldrich), and cholera toxin subunit B (CTB; 1 μg/μl, Invitrogen or Sigma-Aldrich). For the experiments involving proregenerative reagents, 3 µl AAV and other reagents were injected intravitreally by a Hamilton syringe after the removal of an equivalent volume of fluid to prevent outflow of reagents at the injection site.

### Optic nerve trauma model

Optic nerve crush (ONC) surgeries were performed as previously described (Cameron et al., 2020), using cross-action forceps (Dumont, RS-5020, Roboz Surgical Instrument) for 3 s or Dumont #5 forceps for 10 s, 2–3 mm behind the globe. Care was taken to avoid damaging the blood supply to the retina.

### Retinal flat mount and immunohistochemistry

Two weeks after ONC, mice were perfused with saline and then 4% paraformaldehyde (PFA, Sigma-Aldrich) intracardially. Retinal flat mounts were prepared as described previously (Cameron et al., 2020). Briefly, the eyes were harvested and postfixed with 4% PFA for 1 h at room temperature. Retinas were dissected and immunostained with one or more of the following antibodies: anti-RBPMS (1:2,000, custom made, ProSci); anti-βIII tubulin (1:500 rabbit polyclonal, Abcam, ab18207; mouse monoclonal TUJ1, BioLegend, 801202); anti-flag(R) M2 (MilliporeSigma, F1804); anti-CTB (GenWay Biotech, GWB-7B96E4); or anti-CaMKII (Abcam, ab52476) overnight for whole-mount staining or for 1 h for retinal (and optic nerve) sections. Samples were then washed in PBS, TBS (50 mM Tris, 150 mM NaCl), or TBS2T (50 mM Tris, 300 mM NaCl, and 0.1% Tween-20) three times for 1 h and then incubated with a fluorescent secondary antibody (1:200 or 1:500 dilution of stock) for 1 h at room temperature. Secondary antibodies included Alexa 647 goat anti-guinea pig IgG (Thermo Fisher Scientific, A21450), Alexa 647 donkey anti-mouse IgG (Thermo Fisher Scientific, A31571), Alexa 594 donkey anti-rabbit IgG (Thermo Fisher Scientific, A21207), Alexa 594 donkey anti-sheep IgG (Thermo Fisher Scientific, A11016), or Alexa 594 donkey anti-mouse IgG (Thermo Fisher Scientific, A21203). Retinal (and optic nerve) sections were counterstained with DAPI to visualize the cell nuclei. Samples were mounted using the ProLong Gold Antifade mounting medium (Thermo Fisher Scientific) or Fluoromount-G (SouthernBiotech). Images were acquired using a confocal laser scanning microscope (Zeiss 880, Olympus, or Nikon E800 microscope).

### Quantification of CaMKII expression

To measure exogenous CaMKII protein expression, we processed the confocal images using the ImageJ software or native software. The fluorescence intensity in RGCs was quantified by measuring the grayscale value of CaMKII, Flag, βIII tubulin, or RBPMS antibody staining in greater than three cross-sectional or flat-mount images of each retina and subtracting from each cell the mean background intensity based on three randomly selected background regions.

### Quantification of RGC survival and axonal growth

All quantification was performed by investigators masked to condition. For quantification of RGC survival in CaMKII isoform experiments, eight fields (210.23 μm × 210.23 μm) from four quadrants were imaged at 1,200–1,300 μm (middle) and 1,800–2,000 μm (periphery) away from the optic nerve head. For the dose–response experiments, eight fields (397.75 μm × 397.75 μm) from four quadrants were imaged at 1,200–1,300 μm (middle) and 1,800–2,000 μm (periphery) away from the optic nerve head. RBPMS-positive cells were counted manually or in an automated fashion using RGCode (Masin et al., 2021), and the results are presented as cells/mm^2^ for each retina.

Optic nerve axons were traced by CTB delivered by intravitreal injection 1–2 d before killing. For experiments without additional proregenerative treatments, whole optic nerves were surgically explanted, fixed, and cleared using a modified immunolabeling-enabled three-dimensional imaging of solvent-cleared organs (iDISCO) method (Li et al., 2022). Briefly, optic nerves were kept in PBS at 4°C until clearing; passed sequentially through 40, 80, and 100% methanol in 1× PBS solutions for a minimum of 30 min; dichloromethane (DCM)/methanol (2:1) for a minimum of 30 min; 100% DCM for 30 min; and finally dibenzyl ether for 30 s. Cleared nerves were imaged by confocal microscopy (Zeiss 800 or Olympus, 20× air objective) and optically sectioned through the entire optic nerve using the same power and exposure time for all images. Images along the length of the optic nerve were merged across the *z* planes and tiled after acquisition into one composite image containing the crush site and the distal optic nerve. The images were reviewed during acquisition, and for some replicates, the acquisition was not continued distally past where no more regenerating axons were detected, resulting in stored nerve images of different lengths from the crush site. The optic nerves were quantified for axon sprouting or regeneration as described (Li et al., 2022). Briefly, axon numbers were counted at the crossed perpendicular surfaces distal to the crush site at 250, 500, 750, and 1,000 and then every 250 µm until no axons were observed. The first axonal density was estimated at the depths of one-fourth, one-half, and three-fourths of each crossed perpendicular surface. The axonal density was then averaged and multiplied by cross-sectional area. The estimated axon totals were compared among different groups.

In the experiments with proregenerative treatments, surviving RGCs were counted in 6–8 fields of whole-mounted retinas under 200× magnification. After postfixing, optic nerves were equilibrated with 30% sucrose overnight, frozen in O.C.T. Compound (Sakura Finetek USA), and cryosectioned longitudinally at 14 µm. To visualize the regenerating axons, we immunostained the nerve sections with antibodies for either CTB (1:500) or GAP-43 (1:5,000). Axon regeneration was quantified by counting the number of CTB-positive or GAP-43-positive axons at intervals of 0.5 mm distal to the injury site under 200× magnification and converted to total axons at the specified distances as described (Leon et al., 2000).

### Experimental design and statistical analysis

Experiments were performed in a masked fashion as described above. Results are presented as means ± standard error of the mean (SEM). All statistical tests were performed using GraphPad Prism 10. Statistical significance was determined using a two-tailed Mann–Whitney nonparametric test for two groups or using ANOVA for multiple-group comparison, one- or two-way with post hoc testing as indicated. All data are available upon request to the corresponding authors.

## Results

### Expression of CaMKII isoforms in RGCs after intravitreal injection

**Figure 1.**
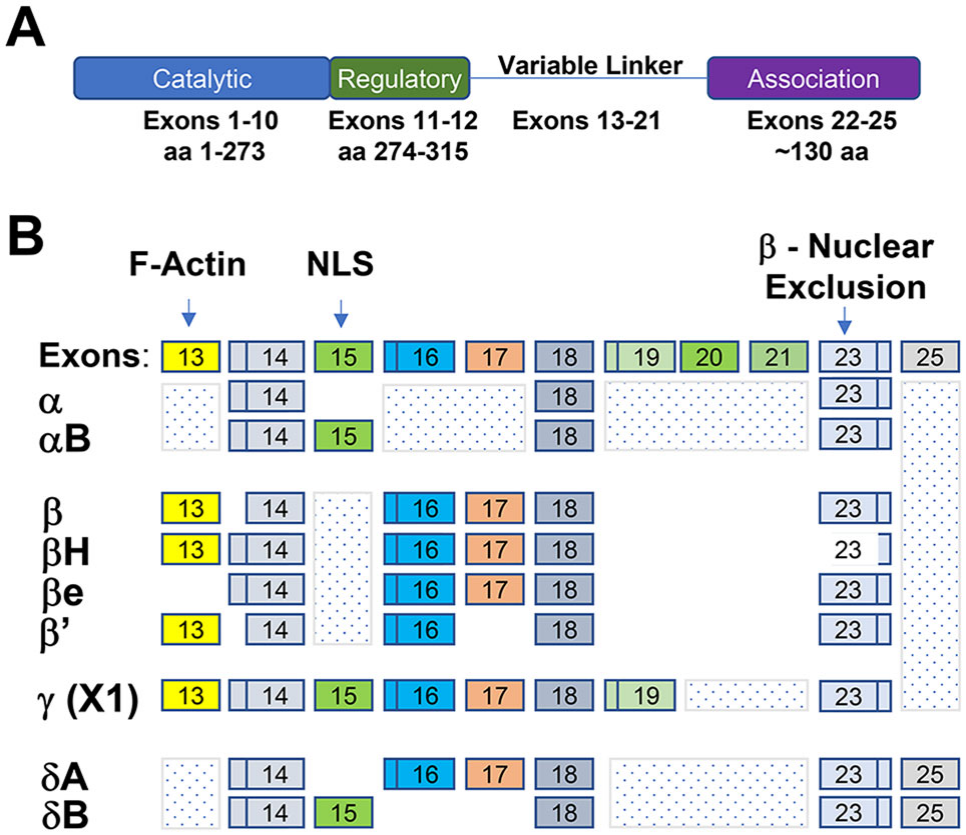
CaMKII structure and variable linker diagram. ***A***, The four mammalian 55–65 kDa CaMKII isoforms α-δ have a conserved domain structure with >75% pairwise identity in the catalytic kinase, regulatory, and association “hub” domains (Sloutsky and Stratton, 2021). ***B***, Alternatively spliced exons of *Camk2a-d* genes and representative isoforms used in our experiments. NLS, nuclear localization sequence.

**Figure 2.**
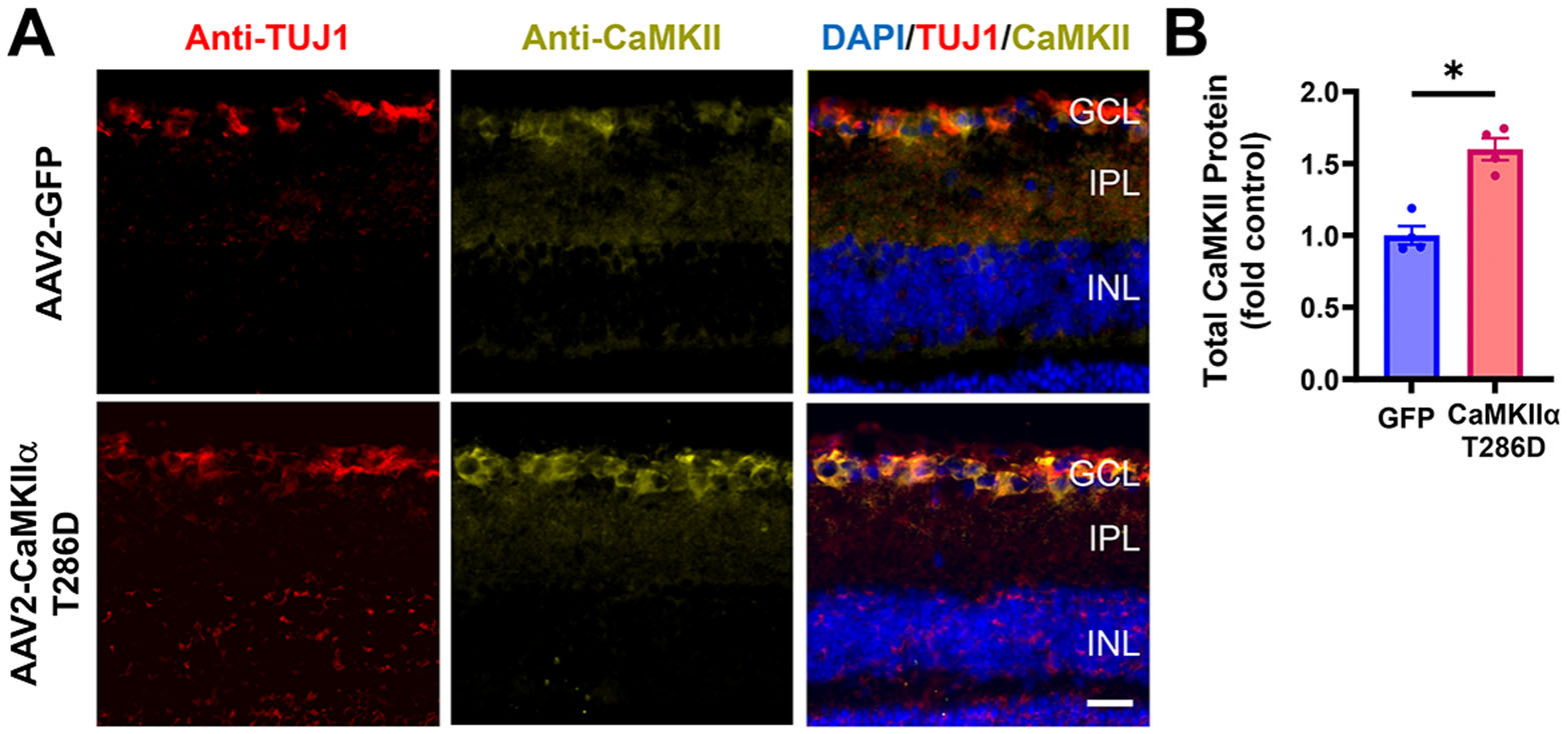
AAV2 mediated CaMKIIα T286D expression increases total CaMKII protein levels in RGCs. AAV2-GFP or AAV2-CaMKIIα T286D (10^11^ vg) was injected intravitreally 2 weeks prior to analysis. ***A***, Retinal cross sections immunostained for the RGC marker TUJ1 (red) and pan-CaMKII (green) and with DAPI for nuclei (blue). Scale bar, 25 μm. GCL, ganglion cell layer; IPL, inner plexiform layer; INL, inner nuclear layer. ***B***, Quantification of CaMKII expression. AAV2-CaMKIIα T286D elevated expression of CaMKII in TUJ1^+^ RGCs. *p < 0.05, *n* = 4 retinas per group, unpaired two-tailed Mann–Whitney test.

CaMKII isoforms are endogenously expressed by RGCs (Guo et al., 2021). All CaMKII family members share a common domain structure and post-translational modifications such as autophosphorylation that can confer Ca^2+^ independence and prolong activation (Bhattacharyya et al., 2020). Here, we studied both the CaMKII α and β isoforms previously described as neuroprotective (Guo et al., 2021), as well as a selection of other isoforms representing all four *Camk2* genes (*a-d*) and a variety of exons from the variable linker region known to contain F-actin binding, nuclear localization, and nuclear exclusion domains (Fig. 1). To test whether the elevation of active CaMKII above physiological levels was sufficient to induce RGC neuroprotection and/or axon regeneration, we first injected AAV2 overexpressing CaMKIIα T286D (10^11^ vg AAV2-CaMKIIα T286D) as previously studied (Guo et al., 2021) into the vitreous of the mouse eye. Two weeks after injection, the fluorescence intensity analysis showed that among βIII tubulin (TUJ1)-positive RGCs, the total CaMKII intensity assayed with a pan-CaMKII primary antibody was increased to 60% with AAV2-CaMKIIα T286D injection compared with controls transduced with AAV2-GFP (Fig. 2). Due to the unavailability of an antibody specifically targeting both CaMKIIα T286D and activated forms of endogenous CaMKII, we were not able to assay for relative levels of activated enzyme, but these data confirm that AAV2-CaMKII transduction increases levels of the total CaMKII expression.

**Figure 3.**
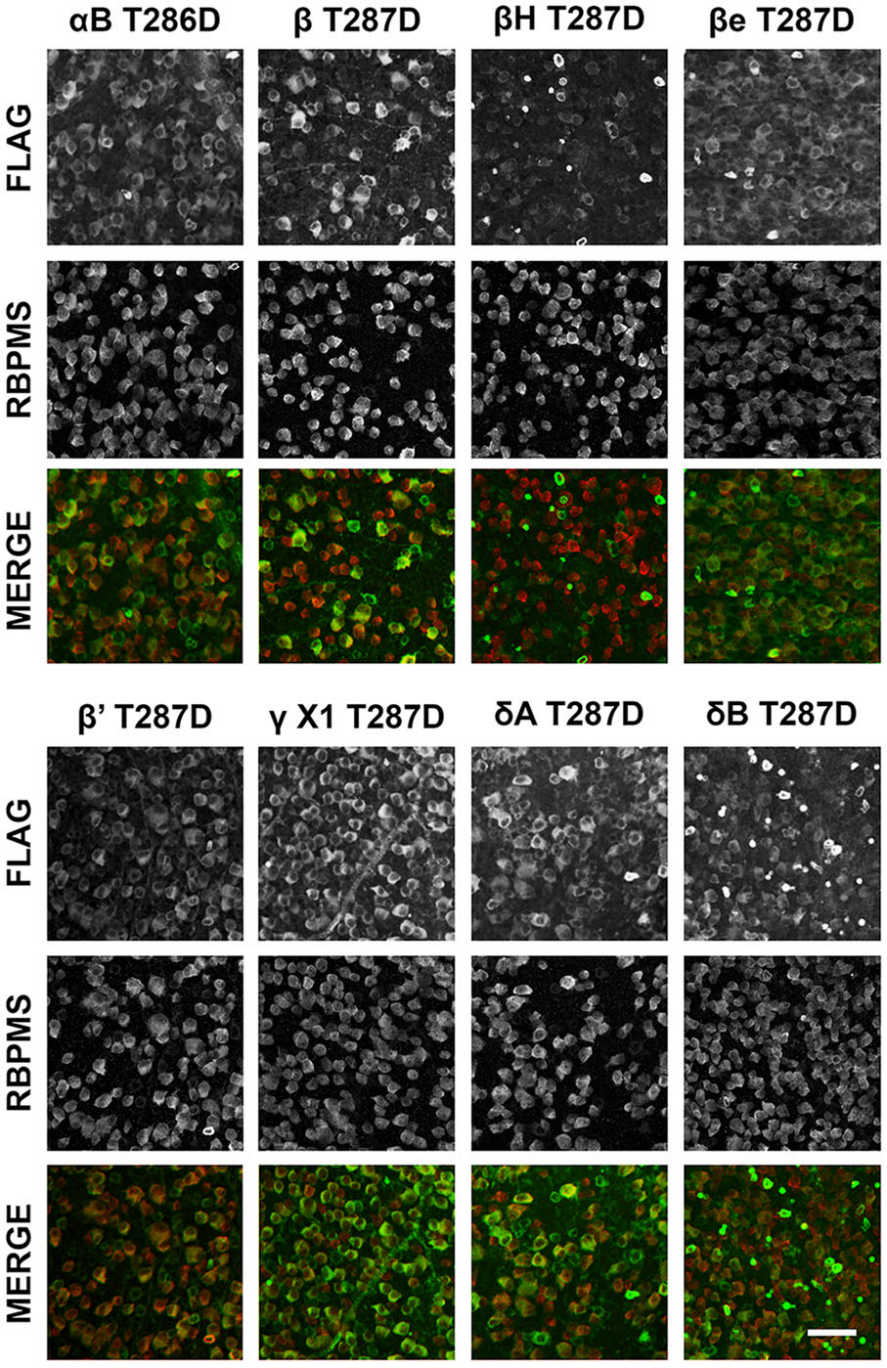
Expression of Flag-tagged constitutively active CaMKII isoforms in RGCs. Flat-mount retinas acquired 2 weeks after intravitreal injection of 4–7 × 10^9^ vg AAV2tm-CaMKII vectors (without injury) were stained with antibodies for the Flag tag (green) and RGC marker RBPMS (red). Flag-tagged protein expression was detectable in most RGCs. Scale bar, 50 μm; *n* = 2 retinas per AAV.

We next explored the expression of other CaMKII isoforms in vivo. Eight newly constructed AAV2tm-CaMKII isoform vectors were injected intravitreally (Fig. 3; ∼4 × 10^9^ vg per eye). To assay the expression of the recombinant proteins, we performed anti-Flag tag immunostaining 2 weeks after administration in the absence of injury. Flag tag staining was detected in nearly all RGCs (albeit less intensely in some cells), evenly distributed across whole-mounted retinas. In addition, despite the use of the *Sncg* promoter, a minority of Flag tag-positive cells were RBPMS negative, likely displaced amacrine cells in the ganglion cell layer. There was some variation in the overall RGC expression level among the isoforms (∼3-fold both between CaMKII isoforms and across biological repeats). All of the isoforms were detected diffusely throughout the RGC, with a generally greater signal outside the nucleus. As all recombinant proteins were successfully expressed in RGCs, all CaMKII isoforms were carried forward into subsequent experiments.

### Overexpression of CaMKII promotes RGC survival after optic nerve injury

**Figure 4.**
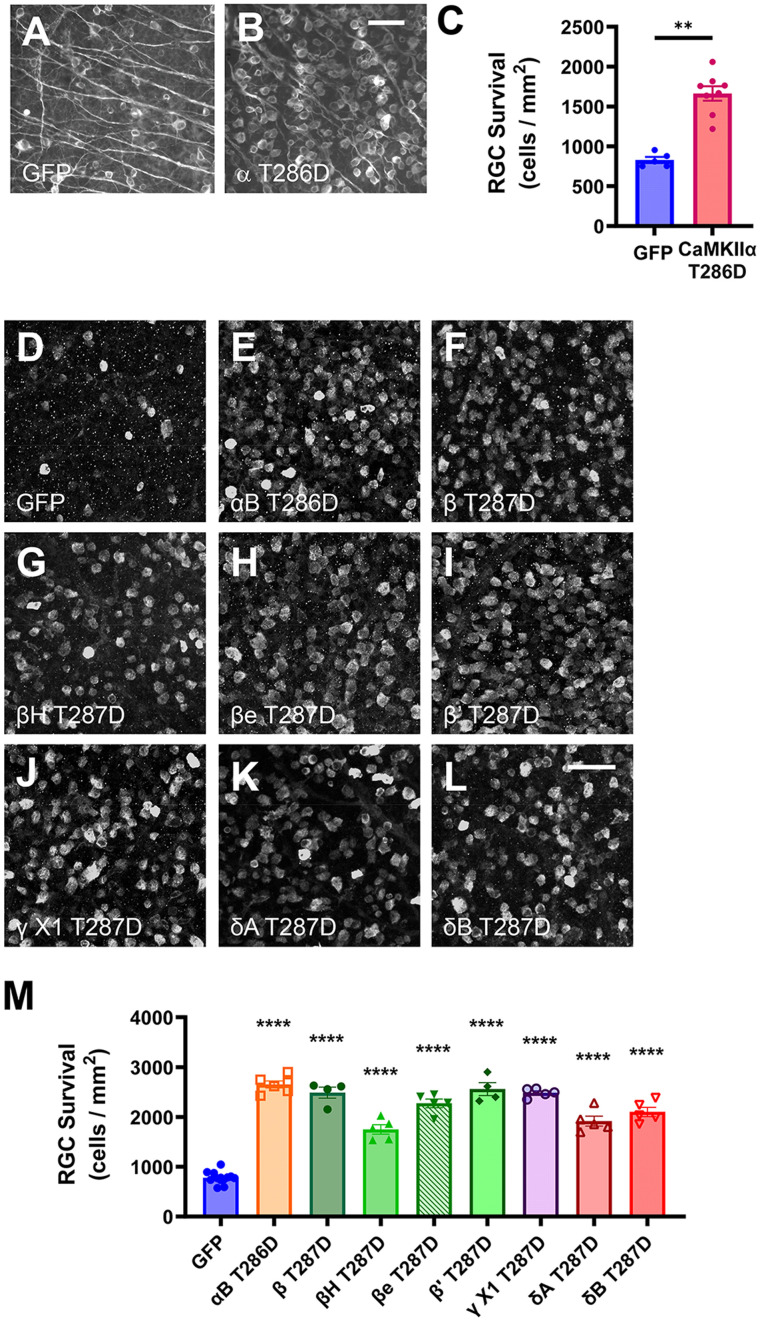
Neuroprotection conferred by constitutively active CaMKII after optic nerve injury. ***A–C***, Either AAV2-GFP or AAV2-CaMKIIα T286D (10^11^ vg) was injected intravitreally 3 weeks prior to crush injury. Two weeks following ONC, RGC survival was quantified by TUJ1 immunostaining (***A***,***B***). Scale bar, 50 μm. AAV2-CaMKIIα T286D increased RGC survival (***C***). **p < 0.01, *n* = 4, eight retinas per group, unpaired two-tailed Mann–Whitney test. ***D***–***M***, AAV2tm-CaMKII T286D or T287D isoforms (4–7 × 10^9^ vg) or GFP control (7 × 10^9^ vg) were injected 2 weeks prior to ONC, followed by RBPMS antibody retinal flat-mount staining (***D–L***; scale bar, 50 μm). Quantification of surviving RGCs showed a significant increase with all CaMKII isoforms tested (***M***). ****p < 0.0001 compared with GFP control, one-way ANOVA with post hoc Dunnett's test.

We first assessed RGC survival after ONC in retinas in which CaMKIIα T286D was overexpressed. 10^11^ vg AAV2-GFP or AAV2-CaMKIIα T286D was intravitreally injected 3 weeks prior to ONC, and retinas were collected 2 weeks after optic nerve injury. Immunostaining for RGCs in fixed retinas and subsequent quantification revealed a significant increase in RGC survival after AAV2-CaMKIIα T286D transduction using TUJ1 as an RGC marker [RGC survival (cells/mm^2^): GFP, 829 ± 39; CaMKIIα T286D, 1,663 ± 92; *p* = 0.002; Fig. 4*A*–*C*]. Although the >80% neuroprotection previously reported for this AAV in C57BL/6J mice was not achieved in this experiment in 129S1 mice, these data support the prior conclusion that the expression of constitutively active CaMKII promotes RGC survival after ONC (Guo et al., 2021).

We next explored whether neuroprotection was similar using a variety of other constitutively activated CaMKII isoforms. AAV2tm vectors were intravitreally injected in 129X1 mice 2 weeks prior to ONC, and retinas were collected 2 weeks after optic nerve injury, followed by RBPMS antibody immunohistochemistry to quantify RGC survival. All of the constitutively active CaMKII isoforms induced significant RGC survival when compared with the survival for AAV2tm-GFP control (Fig. 4*D*–*M*) and noninjured retinas (Fig. 3). Thus, the exogenous expression of constitutively active CaMKII mutants in RGCs is highly neuroprotective after optic nerve trauma, independently of the isoform tested.

### Overexpression of CaMKII suppresses axonal growth after injury

**Figure 5.**
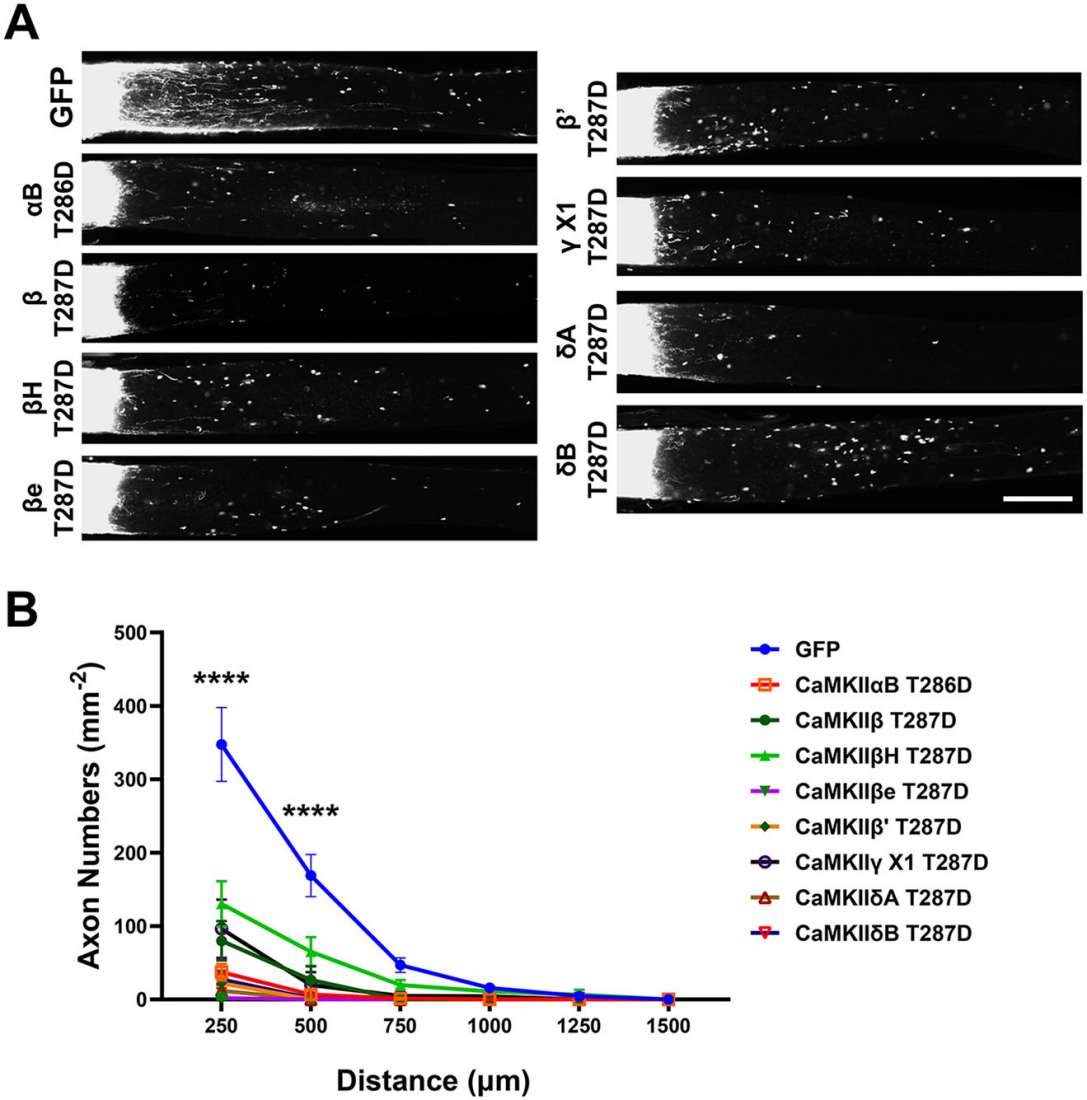
Suppression of axon regeneration conferred by constitutively active CaMKII after optic nerve injury. CTB was injected intravitreally 1–2 d before collecting optic nerve tissue from the mice shown in Figure 4. Optic nerves were cleared using a modified iDISCO method and imaged using confocal microscopy. ***A***, Representative composite tiled images show axonal growth from the crush site on the left. Sprouting axons are visible in the GFP control but were decreased in number after expression of constitutively active CaMKII. Scale bar, 250 µm. ***B***, Quantification of regenerated axons. ****p < 0.0001 for each CaMKII group versus GFP control, *n* = 4–10 per cohort, two-way ANOVA with post hoc Dunnett's test.

**Figure 6.**
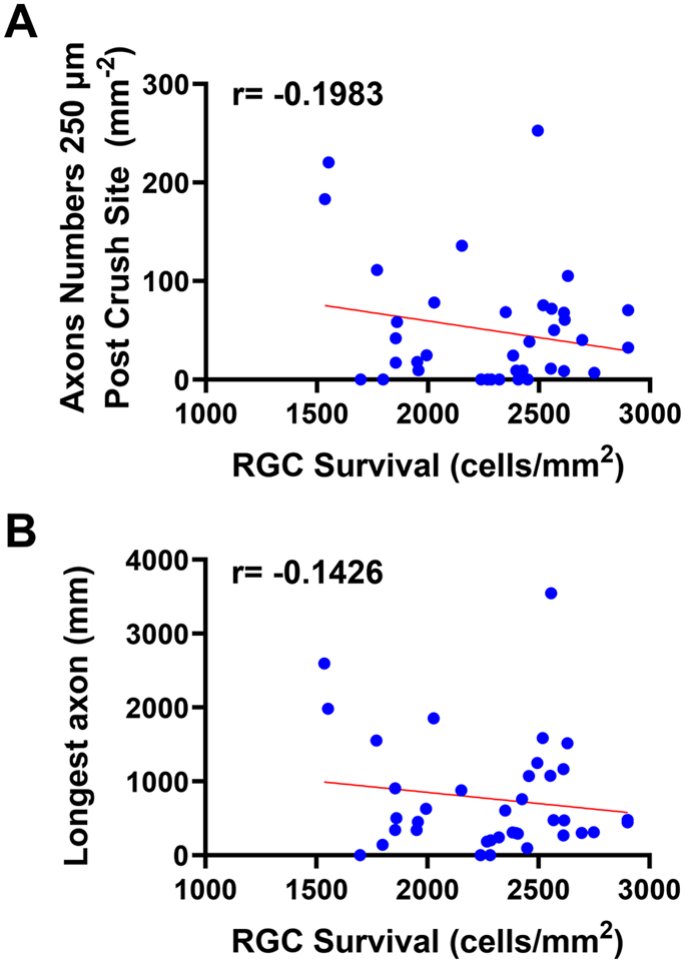
Extent of CaMKII-induced neuroprotection is not correlated with extent of CaMKII-induced axon growth suppression. In these graphs, individual datasets from Figures 4 and 5 are included without distinguishing between CaMKII isoforms, as no isoform selectively conferred neuroprotection and/or axon suppression and as the CaMKII isoforms were all highly overexpressed (see Discussion). ***A***, Pearson’s correlation between RGC survival and axonal density 250 µm away from the injury site. ***B***, Pearson’s correlation between RGC survival and the length of the longest axon.

As axon regeneration is important for the restoration of vision in optic neuropathies, we also determined the effects of constitutively active CaMKII expression on axon growth after optic nerve injury. In the mice studied above for RGC neuroprotection, cholera toxin subunit B was injected intravitreally 2 d before killing to quantify axonal growth. Compared with the short-distance sprouting of axons in the AAV2tm-GFP control condition, we detected even fewer axons sprouting in the proximal optic nerve following the expression of all CaMKII isoforms (Fig. 5). Interestingly, there was not a significant correlation between the survival and axon growth suppression for all CaMKII isoforms whether considered together (Fig. 6) or separately (data not shown), whether measured by the number of proximal sprouting fibers at 250 μm (Fig. 6*A*; *p* = 0.23), or by length of the longest detected regenerating axon (Fig. 6*B*; *p* = 0.39).

**Figure 7.**
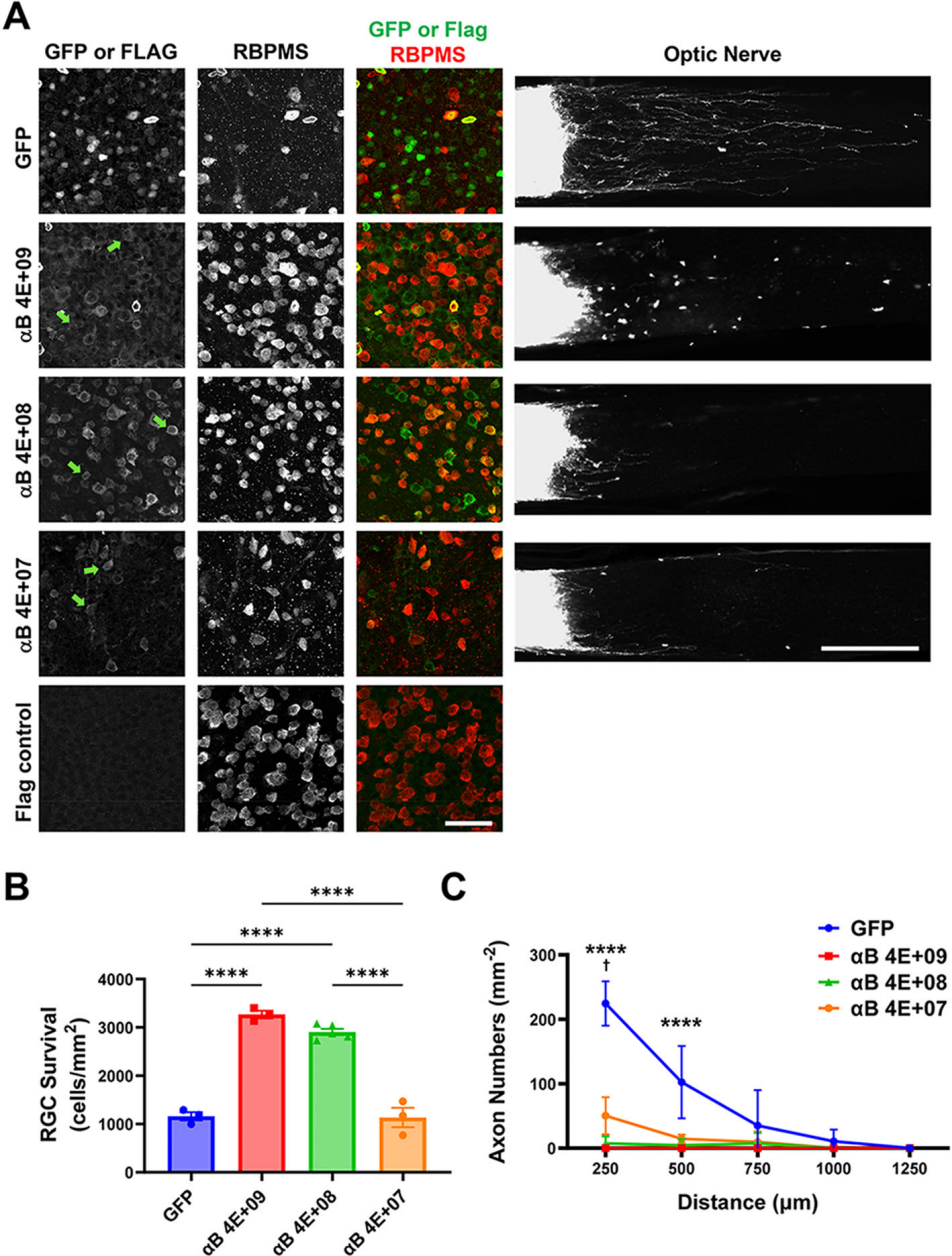
Constitutively active CaMKIIαB is more potent in suppressing axon growth than promoting RGC survival after optic nerve injury. AAV2tm-CaMKII αB T286D (4 × 10^7^–4 × 10^9^ vg) or GFP (7 × 10^9^ vg) control was administered by intravitreal injection 2 weeks before optic nerve injury. Retinas and optic nerves were collected 2 weeks after optic nerve injury. ***A***, Flat-mount retinas stained with Flag antibody or for GFP and RBPMS (left) and CTB-labeled optic nerves (composite tiled images, right). Scale bar, 50 μm for flat mounts, 250 µm for optic nerves. Arrowheads indicated examples of Flag antibody-stained RGCs. The Flag control images show Flag antibody staining for a noncrushed retina with AAV expression of a non-Flag-tagged protein. Flat-mount images were obtained by confocal microscopy with the same acquisition settings. ***B***, Quantification of surviving RGCs. ****p < 0.0001; one-way ANOVA with Tukey’s post hoc testing. ***C***, Quantification of regenerated axons. ****p < 0.0001 compared with GFP control, ^†^*p* < 0.05 for comparison of 4 × 10^9^ and 4 × 10^8^ to 4 × 10^7^ vg αB dose; two-way ANOVA with Tukey’s post hoc testing. *n* = 3–5 per cohort.

To explore the relationship between neuroprotective and axon growth-suppressing effects of CaMKII further, we tested whether decreasing the dose of AAV2tm-CaMKII would still elicit neuroprotection while potentially releasing axons from growth suppression. We repeated the ONC experiment using 4 × 10^9^, 4 × 10^8^, and 4 × 10^7^ vg AAV2tm-CaMKIIαB T286D (Fig. 7), selected as the isoform with the strongest effect on neuroprotection (Fig. 4*M*). As shown by Flag tag staining, decreasing the AAV dose correlated with both the decreased average intensity of Flag tag staining and the number of cells showing staining over the background. At the lowest dose, CaMKIIαB no longer showed any increase in RGC survival but still robustly inhibited axon growth. Thus, diverse CaMKII isoforms strongly inhibit axon growth after optic nerve injury and, at least for CaMKIIαB, the T286D mutation appears to inhibit axon growth with more potency than to induce neuroprotection.

### AAV2-CaMKIIα T286D suppression of axon regeneration is dominant over previously reported proregenerative treatments

**Figure 8.**
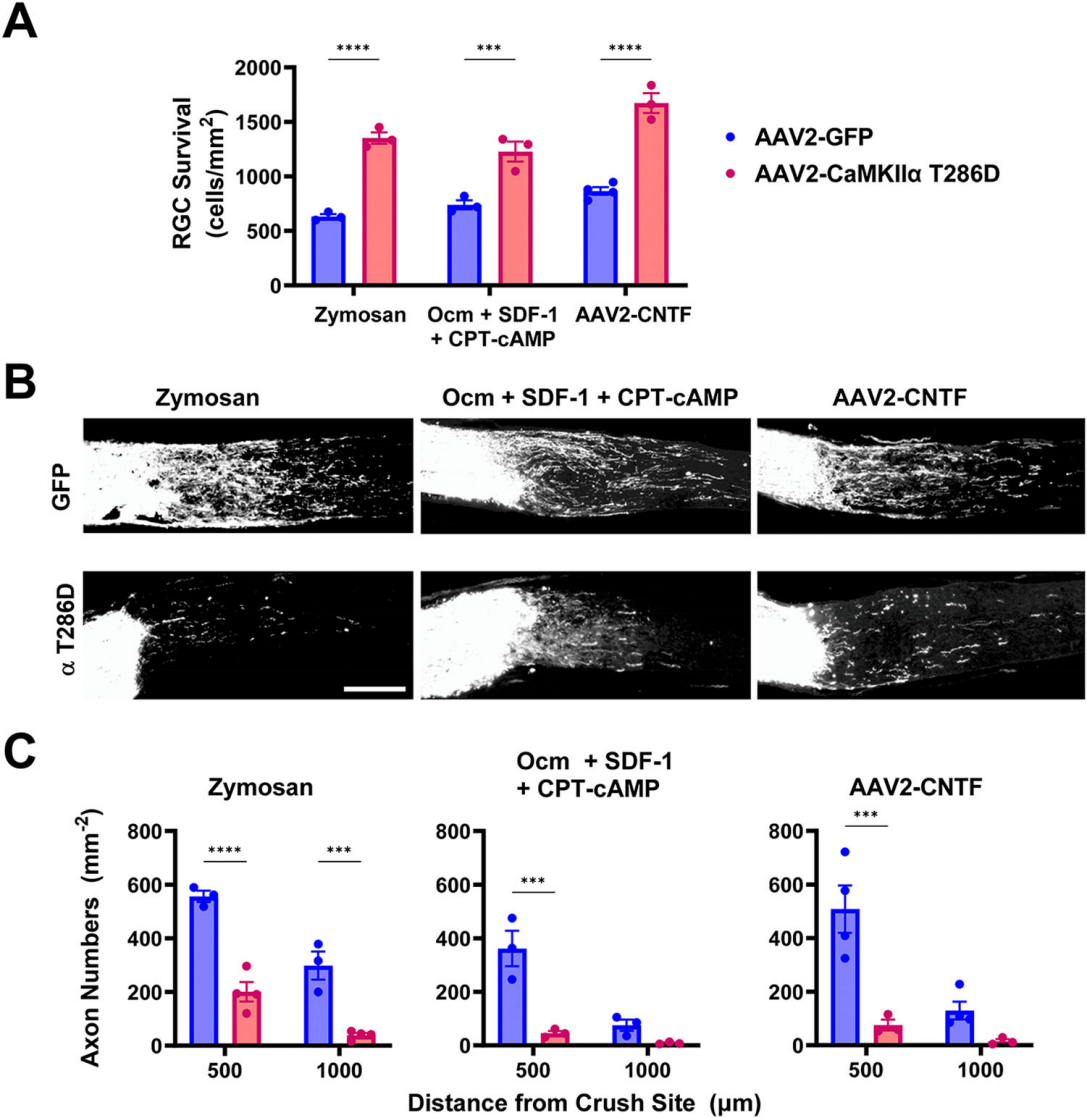
CaMKIIα T286D expression promotes RGC survival but suppresses the effects of several proregenerative treatments. 10^11^ vg AAV2-GFP (blue datapoints) or AAV2-CaMKIIα T286D (red datapoints) was intravitreally injected 3 weeks prior to ONC, followed by AAV2-CNTF injection 2 weeks prior to ONC or zymosan or Ocm + SDF-1 + CPT-cAMP treatment at the time of ONC. Two weeks following ONC, RGC survival and axon regeneration were quantified. ***A***, Quantification of RGC survival. In each proregenerative treatment group, CaMKIIα T286D expression promoted RGC survival. ***p < 0.001, ****p < 0.0001; two-way ANOVA with Sidak's post hoc testing. ***B***, Regenerating axons were detected by CTB immunohistochemistry (composite tiled images shown; scale bar, 200 μm). ***C***, Quantification of RGC axon regeneration. Within each proregenerative treatment group, CaMKIIα T286D expression diminished RGC axon regeneration after a crush injury. For both ***A*** and ***C***: ***p < 0.001, ****p < 0.0001; *n* = 3–4 per group; two-way ANOVA with Sidak's post hoc testing.

To determine whether CaMKII-mediated axon growth suppression would occur even in the presence of known proregenerative interventions, we combined 10^11^ vg AAV2-GFP or AAV2-CaMKIIα T286D injection 3 weeks prior to ONC in 129S1 mice with AAV2-CNTF injection 2 weeks prior to ONC (Leaver et al., 2006; Xie et al., 2021) or with zymosan (Leon et al., 2000; Yin et al., 2003) or Ocm + SDF-1 + CPT-cAMP (Xie et al., 2022) treatment at the time of ONC. We found that AAV2-CaMKIIα T286D increased RGC survival over any of the three additional treatments (Fig. 8*A*) but strongly suppressed the axon regeneration induced by any of these, as measured at 500 or 1,000 μm from the injury site (Fig. 8*B*,*C*).

## Discussion

The results of our study confirm the remarkable neuroprotective effects of constitutively active CaMKII in RGCs yet demonstrate that increased CaMKII activity suppresses optic nerve axon regeneration induced by a variety of agents. These results directly impact the decisions as to whether the CaMKII signaling pathway should be targeted in the treatment of optic neuropathies such as the highly prevalent disease glaucoma. Inhibiting RGC loss is the main goal in preventing the progression of optic neuropathies. However, the replacement and/or rescue of injured RGCs and the subsequent reconnection of axons to the brain will be central to the treatment of vision loss often present in patients at diagnosis. That RGC expression of constitutively active CaMKII has the observed opposing effects in vivo complicates the development of CaMKII-enhancing therapies in optic neuropathy.

Both the findings of the Chen lab (Guo et al., 2021) and now our independently developed data demonstrate that the neuroprotection conferred by AAV-based expression of active CaMKII greatly surpasses other previously identified interventions, with numbers of surviving RGCs almost reaching the normal levels in healthy mouse retinas. Thus, we can now consider CaMKII a leading target for therapeutic development in terms of arresting the progression of optic neuropathies, especially since similar findings have been replicated across three different institutions [Mount Sinai Health System (Guo et al., 2021) and herein Boston Children's Hospital and Stanford University], using three different mouse strains [C57BL/6J (Guo et al., 2021), 129X1/SvJ, and 129S1] and using AAV vectors with different capsids and promoters.

Interestingly, the role of CaMKII in RGC neuroprotection is in contrast to that for CaMKII in ischemic CNS stroke. The overall inhibition of CaMKII activity can be beneficial in stroke, and CaMKII inhibitors have been proposed as therapeutics promoting neuroprotection in cerebrovascular disease (Deng et al., 2017; Zhang et al., 2021). Taken together, these results highlight the importance of localized (vs systemic) CaMKII therapy if a CaMKII gene therapy is to be deployed in chronic ophthalmic diseases such as glaucoma.

Surprisingly, upon examination of the corresponding optic nerves, we found that axon regeneration was highly suppressed by the expression of constitutively active CaMKII. These results are reminiscent of the effects of a constitutively active truncated CaMKII, but not a constitutively active form of CaMKIV in preventing axon outgrowth by spiral ganglion neurons in vitro, cells in which both kinases promote survival (Hansen et al., 2003; Takemoto-Kimura et al., 2017). Our finding that neuroprotective efficacy did not correlate with the extent of regenerative suppression, combined with our finding that the suppression of axon regeneration was nearly complete at a 10-fold lower dose of AAV2tm-CaMKIIαB than at which neuroprotection was detected, suggests that CaMKII has different targets for regulating survival versus axon growth.

Dose-dependent differences in CaMKII functionality may be due to the differential incorporation of exogenous constitutively active enzyme into endogenous dodecamers containing different CaMKII isoforms and/or due to preferential subcellular localization into signaling complexes specific for axon suppression at lower levels of expression. These hypotheses will be explored in future research.

CaMKII is a multifunctional protein kinase with diverse substrates including ion channels, signaling enzymes, metabolic enzymes, cytoskeletal proteins, transcription factors, and histone deacetylases (Braun and Schulman, 1995; Yasuda et al., 2022). As most RGCs in the AAV-treated eyes appeared to express recombinant CaMKII protein, albeit some at very low levels, RGC autonomous signaling presumably contributed to the neuroprotective and axon-suppressive effects of CaMKII, although in principle we cannot exclude the effects of AAV-mediated CaMKII expression in cell types other than RGCs, such as displaced amacrine cells. In prior work on RGC neuroprotection in vivo (Guo et al., 2021), the transcription factor CREB was implicated as necessary for CaMKIIα's neuroprotective effect, although the role of CREB in RGC neuroprotection is controversial (Pita-Thomas et al., 2021). Another potentially relevant CaMKII effector is the transcription factor nuclear factor-κB (NF-κB; Dvoriantchikova et al., 2016), as suggested by studies showing that the in vitro neuroprotective effects of CaMKIIγ and CaMKIIδ during oxygen/glucose deprivation and reoxygenation correlated with the activation of the NF-κB pathway (Ye et al., 2019).

CREB is not likely the CaMKII effector responsible for the observed RGC axon growth suppression, as CaMKII inhibition blocked RGC axon outgrowth in vitro stimulated by oncomodulin and elevated cAMP, a treatment that also activates CREB (Yin et al., 2006). Likewise, other targets of CaMKII signaling that have been implicated in the regulation of axon growth seem unlikely to be responsible for the axon growth suppression. For example, via PSMC5 phosphorylation, CaMKIIα downregulates AMPK in dorsal root ganglion axons, stimulating axon growth after spinal cord injury (Kong et al., 2020). It is not clear how activated CaMKII might repress axon growth after optic nerve injury, but we hypothesize that AAV-based expression of constitutively active CaMKII in RGCs resulted in the broad activation of CaMKII-dependent signaling pathways throughout the neuron, including those unnecessary for neuroprotection that can repress axon regeneration. Interestingly, CaMKIIβ T287D mutation inhibits the isoform's ability to bind actin, decreasing dendritic arborization in hippocampal neurons (Fink et al., 2003). As CaMKII forms heterododecamers and as CaMKII holoenzyme subunits activate each other in trans, it is possible that the expression of constitutively active CaMKII protein kinases in RGCs affected endogenous CaMKIIβ activity to regulate axon regeneration.

Accordingly, due to CaMKII heterododecamerization, overexpressed alternatively spliced CaMKII isoforms were likely incorporated into different CaMKII holoenzymes present within different intracellular compartments within which the activated isoforms may not normally be substantively represented. CaMKII is kinetically slow, and regulated binding to compartmentalized anchoring proteins or substrates (e.g., dendritic glutamate receptors) is important for CaMKII function (Yasuda et al., 2022; Rostas and Skelding, 2023). Notably, alternative splicing results in differential intracellular CaMKII localization. Using the nomenclature proposed by Sloutsky and Stratton, α, γ, and δ isoforms with exon 15 are localized to the nucleus, whereas β and γ exon 13 confers F-actin binding (Fig. 1; Sloutsky and Stratton, 2021). β isoforms with a shorter exon 23 (H and eH) are also enriched in the nucleus (Cook et al., 2018). In the survey of different CaMKII isoforms, we expressed the exogenous kinases using AAV doses similar to those reported by the Chen lab (Guo et al., 2021), doses subsequently found to be much higher than necessary for the effects of AAV2tm-CaMKIIαB. As a result, similar to the Chen lab study, individual recombinant isoforms were expressed at a level greater than the combined level of all RGC CaMKII protein, and we did not detect the expected nuclear enrichment of previously reported nuclear isoforms, including αB at the lowest dose tested. This level of overexpression may account for the similar neuroprotective and axon-suppressive effects of all of the CaMKII isoforms, as it is reasonable to assume that the overexpressed proteins activated all CaMKII compartments within the RGC nonspecifically. Further research addressing isoform biological specificity and subcellular localization in RGCs will be necessary to dissect the potentially different roles of CaMKII isoforms in neuroprotection and axon suppression.

Limitations of this study include that neuroprotection and axon growth were studied only for 2 weeks after injury. Longer longitudinal studies will be required to assess the potential significance of this potential therapy, although the Chen lab has demonstrated the beneficial effects of CaMKIIα gene therapy for 6 and 12 months after crush and excitotoxic injury, respectively (Guo et al., 2021). In addition, we studied CaMKII overexpression in combination with a series of proregenerative treatments, including both direct activation of an intracellular signaling pathway (CPT-cAMP) and modulation of intracellular signaling pathways by extracellular stimuli [CNTF gene therapy, which acts via chemokine CCL5 (Xie et al., 2021), Ocm, zymosan, SDF-1]. It will be important to understand whether CaMKII treatment is effective in the context of additional proregenerative treatments, including in PTEN or KLF9 gene deletion or Oct4, Sox2, or Klf4 overexpression (Park et al., 2008; Moore et al., 2009; Lu et al., 2020), among others. Understanding the underlying mechanisms of CaMKII action in RGCs will be key to deriving strategies that promote RGC survival without compromising axon regeneration, an important goal in light of the profound neuroprotection that may be provided by CaMKII gene therapy in optic neuropathies.

## Synthesis

Reviewing Editor: Lynda Erskine, University of Aberdeen Decisions are customarily a result of the Reviewing Editor and the peer reviewers coming together and discussing their recommendations until a consensus is reached. When revisions are invited, a fact-based synthesis statement explaining their decision and outlining what is needed to prepare a revision will be listed below. The following reviewer(s) agreed to reveal their identity: NONE. Note: If this manuscript was transferred from JNeurosci and a decision was made to accept the manuscript without peer review, a brief statement to this effect will instead be what is listed below.

As you will see from the review synthesis below, the referees are enthusiastic about your work and we would like to published a revised version if the referees' comments can be satisfactorily addressed.

General comments from Reviewers:

Developing protective strategies to protect retinal ganglion cells (RGCs) should be useful for glaucoma and other optic neuropathies. In this manuscript, the authors present their results showing that over-expression of CaMKII in RGCs could protect these cells from degeneration following optic nerve crush (ONC). However, this treatment also suppresses axon regrowth spontaneously occurred after injury or axon regeneration induced by different means such as CNTF or zymosan. Although protective effects of CaMKII has been recently reported by Guo et al., 2021, its effect on blocking different forms of axon regrowth is novel. These results are important, for revealing complex nature of neuroprotection.

In their submission, Xia and colleagues expand upon the recent finding from Bo Chen's lab that CaMKII activation can very strongly protect RGCs from a number of different degenerative stimuli. Using the optic nerve crush model, first, a range of different CaMKII constitutively active constructs all demonstrate significant protection of RGCs. Intriguingly, co-treatment with a range of axon regenerative treatments are rendered futile with CaMKII treatments. Overall, these are important contributions to the field. However, there are a number of issues that need to be addressed.

Major points to address:

1. A critical claim from this study is that the dosage of CaMKII activity that prevents axon regeneration is lower than that required for preventing neurodegeneration. However, there is no demonstration that the low titer AAV treatment is even transfecting RGCs and expressing protein. The full titer treatment indicated that only ~60% more CaMKIIa is present by immunostaining with this treatment. There is no apparent Flag immunostaining. And lower AAV titers often lead to both a reduced expression level and a lower number of cells that are transfected in the first place. Some approach (e.g. tyramide amplification, in situ hybridization) to show the presence of this treatment is required to demonstrate any gene expression.

2. While it is an important observation that 3 different well-characterized regenerative treatments are inhibited by CaMKIIa treatment, these treatments all represent extrinsic signaling that stimulates regenerative pathways. It would be extremely interesting to also examine PTEN knockout/knockdown as this manipulation acts downstream of internal signaling pathways. Performing this experiment in the PTEN KO would determine if CaMKIIa completely inhibits axon regeneration, or perturbs cell signaling pathways that are regenerative.

3. How long can CaMKII-expressed RGCs survive after optic nerve injury? This will be informative as to the translational potential of this strategy.

4. Student's T-tests were used for comparisons to controls. T-test assumes normal distribution, which cannot be determined with small sample numbers. Rank Sum/Mann Whitney U Test is appropriate for 2 group comparisons where normal distribution cannot be confirmed.

Other points:

1. Page 13 paragraph 2 "...quantification revealed a two-fold increase..." This data does not indicate a precisely 2-fold increase. Please calculate the true increase and indicate variance.

2. Same paragraph "These data confirm previous work on RGC survival...(Guo et al.)" In Guo et al similar treatments lead to roughly 3-4 fold increases in survival. Perhaps worth indicating discrepancy.

3. Page 13 paragraph 3 "All of the constitutively active CaMKII isoforms showed robust RGC survival...nearly to the level of 100% RGC neuroprotection (about 3,000 RGCs/mm2)". First, where is the 3,000 RGCs/mm2 number derived from. There is no reference or primary data indicating this density. Second, "nearly to the level of 100%" is meaningless and a stretch. Taking the 3,000 number as 100% and estimating the values from 4M, the treatments range from ~60-85% survival rates. Please use more precise wording here, or detailed values.

4. Figure 3. Some of these constructs look much more expressed in amacrine cells than others (BH T287D and gB T287D). Some mention of this would be worthwhile.

-AAV titers for AAV2-CaMKIIa T286D are not provided for Figure 8. Critical for interpreting these data.

5. Throughout. There are amacrine cells expressing AAV carrying transgenes throughout the paper despite using the "RGC selective" Sncg promoter. This needs to be pointed out as the observation has important implications for future experiments that assume this technique confers RGC specificity.

## References

[B1] Apara A, Goldberg JL (2014) Molecular mechanisms of the suppression of axon regeneration by KLF transcription factors. Neural Regen Res 9:1418–1421. 10.4103/1673-5374.139454 25317150 PMC4192940

[B2] Baldicano AK, Nasir-Ahmad S, Novelli M, Lee SCS, Do MTH, Martin PR, Grunert U (2022) Retinal ganglion cells expressing CaM kinase II in human and nonhuman primates. J Comp Neurol 530:1470–1493. 10.1002/cne.25292 35029299 PMC9010361

[B3] Bhattacharyya M, Karandur D, Kuriyan J (2020) Structural insights into the regulation of Ca(2+)/calmodulin-dependent protein kinase II (CaMKII). Cold Spring Harb Perspect Biol 12:a035147. 10.1101/cshperspect.a035147 31653643 PMC7263085

[B4] Boczek T, et al. (2019) Regulation of neuronal survival and axon growth by a perinuclear cAMP compartment. J Neurosci 39:5466–5480. 10.1523/JNEUROSCI.2752-18.2019 31097623 PMC6616289

[B5] Braun AP, Schulman H (1995) The multifunctional calcium/calmodulin-dependent protein kinase: from form to function. Annu Rev Physiol 57:417–445. 10.1146/annurev.ph.57.030195.002221 7778873

[B6] Cameron EG, Xia X, Galvao J, Ashouri M, Kapiloff MS, Goldberg JL (2020) Optic nerve crush in mice to study retinal ganglion cell survival and regeneration. Bio Protoc 10:e3559. 10.21769/BioProtoc.3559 PMC7197875 32368566

[B7] Chang KC, et al. (2021) Posttranslational modification of Sox11 regulates RGC survival and axon regeneration. eNeuro 8:ENEURO.0358-20.2020. 10.1523/ENEURO.0358-20.2020. PMC7890524 33441400

[B8] Chang EE, Goldberg JL (2012) Glaucoma 2.0: neuroprotection, neuroregeneration, neuroenhancement. Ophthalmology 119:979–986. 10.1016/j.ophtha.2011.11.003 22349567 PMC3343191

[B9] Cheng Y, et al. (2022) Transcription factor network analysis identifies REST/NRSF as an intrinsic regulator of CNS regeneration in mice. Nat Commun 13:4418. 10.1038/s41467-022-31960-7 35906210 PMC9338053

[B10] Cook SG, et al. (2018) Analysis of the CaMKIIalpha and beta splice-variant distribution among brain regions reveals isoform-specific differences in holoenzyme formation. Sci Rep 8:5448. 10.1038/s41598-018-23779-4 29615706 PMC5882894

[B11] Deng G, Orfila JE, Dietz RM, Moreno-Garcia M, Rodgers KM, Coultrap SJ, Quillinan N, Traystman RJ, Bayer KU, Herson PS (2017) Autonomous CaMKII activity as a drug target for histological and functional neuroprotection after resuscitation from cardiac arrest. Cell Rep 18:1109–1117. 10.1016/j.celrep.2017.01.011 28147268 PMC5540152

[B12] Dvoriantchikova G, Pappas S, Luo X, Ribeiro M, Danek D, Pelaez D, Park KK, Ivanov D (2016) Virally delivered, constitutively active NFkappaB improves survival of injured retinal ganglion cells. Eur J Neurosci 44:2935–2943. 10.1111/ejn.13383 27564592 PMC5138106

[B13] Fan W, Li X, Cooper NG (2007) CaMKIIalphaB mediates a survival response in retinal ganglion cells subjected to a glutamate stimulus. Invest Ophthalmol Vis Sci 48:3854–3863. 10.1167/iovs.06-1382 17652761

[B14] Fink CC, Bayer KU, Myers JW, Ferrell JE Jr, Schulman H, Meyer T (2003) Selective regulation of neurite extension and synapse formation by the beta but not the alpha isoform of CaMKII. Neuron 39:283–297. 10.1016/S0896-6273(03)00428-8 12873385

[B15] Galvao J, Iwao K, Apara A, Wang Y, Ashouri M, Shah TN, Blackmore M, Kunzevitzky NJ, Moore DL, Goldberg JL (2018) The Kruppel-like factor gene target Dusp14 regulates axon growth and regeneration. Invest Ophthalmol Vis Sci 59:2736–2747. 10.1167/iovs.17-23319 29860460 PMC5983061

[B16] Guo X, et al. (2021) Preservation of vision after CaMKII-mediated protection of retinal ganglion cells. Cell 184:4299–4314.e12. 10.1016/j.cell.2021.06.031 34297923 PMC8530265

[B17] Hansen MR, Bok J, Devaiah AK, Zha XM, Green SH (2003) Ca2+/calmodulin-dependent protein kinases II and IV both promote survival but differ in their effects on axon growth in spiral ganglion neurons. J Neurosci Res 72:169–184. 10.1002/jnr.10551 12671991

[B18] Kong G, et al. (2020) AMPK controls the axonal regenerative ability of dorsal root ganglia sensory neurons after spinal cord injury. Nat Metab 2:918–933. 10.1038/s42255-020-0252-3 32778834

[B19] Kunzevitzky NJ, Almeida MV, Goldberg JL (2010) Amacrine cell gene expression and survival signaling: differences from neighboring retinal ganglion cells. Invest Ophthalmol Vis Sci 51:3800–3812. 10.1167/iovs.09-4540 20445109 PMC2904021

[B20] Leaver SG, Cui Q, Plant GW, Arulpragasam A, Hisheh S, Verhaagen J, Harvey AR (2006) AAV-mediated expression of CNTF promotes long-term survival and regeneration of adult rat retinal ganglion cells. Gene Ther 13:1328–1341. 10.1038/sj.gt.3302791 16708079

[B21] Leon S, Yin Y, Nguyen J, Irwin N, Benowitz LI (2000) Lens injury stimulates axon regeneration in the mature rat optic nerve. J Neurosci 20:4615–4626. 10.1523/JNEUROSCI.20-12-04615.2000 10844031 PMC6772462

[B22] Li L, et al. (2022) Single-cell transcriptome analysis of regenerating RGCs reveals potent glaucoma neural repair genes. Neuron 110:2646–2663.e6. 10.1016/j.neuron.2022.06.022 35952672 PMC9391304

[B23] Lorber B, Howe ML, Benowitz LI, Irwin N (2009) Mst3b, an Ste20-like kinase, regulates axon regeneration in mature CNS and PNS pathways. Nat Neurosci 12:1407–1414. 10.1038/nn.2414 19855390 PMC2770175

[B24] Lu Y, et al. (2020) Reprogramming to recover youthful epigenetic information and restore vision. Nature 588:124–129. 10.1038/s41586-020-2975-4 33268865 PMC7752134

[B25] Masin L, Claes M, Bergmans S, Cools L, Andries L, Davis BM, Moons L, De Groef L (2021) A novel retinal ganglion cell quantification tool based on deep learning. Sci Rep 11:702. 10.1038/s41598-020-80308-y 33436866 PMC7804414

[B26] Moore DL, Blackmore MG, Hu Y, Kaestner KH, Bixby JL, Lemmon VP, Goldberg JL (2009) KLF family members regulate intrinsic axon regeneration ability. Science 326:298–301. 10.1126/science.1175737 19815778 PMC2882032

[B27] Park KK, et al. (2008) Promoting axon regeneration in the adult CNS by modulation of the PTEN/mTOR pathway. Science 322:963–966. 10.1126/science.1161566 18988856 PMC2652400

[B28] Petrs-Silva H, et al. (2011) Novel properties of tyrosine-mutant AAV2 vectors in the mouse retina. Mol Ther 19:293–301. 10.1038/mt.2010.234 21045809 PMC3034844

[B29] Pita-Thomas W, Goncalves TM, Kumar A, Zhao G, Cavalli V (2021) Genome-wide chromatin accessibility analyses provide a map for enhancing optic nerve regeneration. Sci Rep 11:14924. 10.1038/s41598-021-94341-y 34290335 PMC8295311

[B30] Rostas JAP, Skelding KA (2023) Calcium/calmodulin-stimulated protein kinase II (CaMKII): different functional outcomes from activation, depending on the cellular microenvironment. Cells 12:401. 10.3390/cells12030401 36766743 PMC9913510

[B31] Sloutsky R, Stratton MM (2021) Functional implications of CaMKII alternative splicing. Eur J Neurosci 54:6780–6794. 10.1111/ejn.14761 32343011 PMC7836398

[B32] Takemoto-Kimura S, Suzuki K, Horigane SI, Kamijo S, Inoue M, Sakamoto M, Fujii H, Bito H (2017) Calmodulin kinases: essential regulators in health and disease. J Neurochem 141:808–818. 10.1111/jnc.14020 28295333

[B33] Terashima T, Ochiishi T, Yamauchi T (1994) Immunocytochemical localization of calcium/calmodulin-dependent protein kinase II isoforms in the ganglion cells of the rat retina: immunofluorescence histochemistry combined with a fluorescent retrograde tracer. Brain Res 650:133–139. 10.1016/0006-8993(94)90215-1 7953663

[B34] Tetenborg S, Yadav SC, Hormuzdi SG, Monyer H, Janssen-Bienhold U, Dedek K (2017) Differential distribution of retinal Ca(2+)/calmodulin-dependent kinase II (CaMKII) isoforms indicates CaMKII-beta and -delta as specific elements of electrical synapses made of connexin36 (Cx36). Front Mol Neurosci 10:425. 10.3389/fnmol.2017.00425 29311815 PMC5742114

[B35] Wang Q, et al. (2020) Mouse gamma-synuclein promoter-mediated gene expression and editing in mammalian retinal ganglion cells. J Neurosci 40:3896–3914. 10.1523/JNEUROSCI.0102-20.2020 32300046 PMC7219295

[B36] Watkins TA, Wang B, Huntwork-Rodriguez S, Yang J, Jiang Z, Eastham-Anderson J, Modrusan Z, Kaminker JS, Tessier-Lavigne M, Lewcock JW (2013) DLK initiates a transcriptional program that couples apoptotic and regenerative responses to axonal injury. Proc Natl Acad Sci U S A 110:4039–4044. 10.1073/pnas.1211074110 23431164 PMC3593899

[B37] Xie L, et al. (2022) Monocyte-derived SDF1 supports optic nerve regeneration and alters retinal ganglion cells’ response to Pten deletion. Proc Natl Acad Sci U S A 119:e2113751119. 10.1073/pnas.2113751119 35394873 PMC9169637

[B38] Xie L, Yin Y, Benowitz L (2021) Chemokine CCL5 promotes robust optic nerve regeneration and mediates many of the effects of CNTF gene therapy. Proc Natl Acad Sci U S A 118:e2017282118. 10.1073/pnas.2017282118 33627402 PMC7936361

[B39] Yasuda R, Hayashi Y, Hell JW (2022) CaMKII: a central molecular organizer of synaptic plasticity, learning and memory. Nat Rev Neurosci 23:666–682. 10.1038/s41583-022-00624-2 36056211

[B40] Ye J, et al. (2019) Ischemic injury-induced CaMKIIdelta and CaMKIIgamma confer neuroprotection through the NF-kappaB signaling pathway. Mol Neurobiol 56:2123–2136. 10.1007/s12035-018-1198-2 29992531 PMC6394630

[B41] Yin Y, Cui Q, Li Y, Irwin N, Fischer D, Harvey AR, Benowitz LI (2003) Macrophage-derived factors stimulate optic nerve regeneration. J Neurosci 23:2284–2293. 10.1523/JNEUROSCI.23-06-02284.2003 12657687 PMC6742044

[B42] Yin Y, Henzl MT, Lorber B, Nakazawa T, Thomas TT, Jiang F, Langer R, Benowitz LI (2006) Oncomodulin is a macrophage-derived signal for axon regeneration in retinal ganglion cells. Nat Neurosci 9:843–852. 10.1038/nn1701 16699509

[B43] Zhang X, Connelly J, Levitan ES, Sun D, Wang JQ (2021) Calcium/calmodulin-dependent protein kinase II in cerebrovascular diseases. Transl Stroke Res 12:513–529. 10.1007/s12975-021-00901-9 33713030 PMC8213567

